# Partial Reprogramming of Pluripotent Stem Cell-Derived Cardiomyocytes into Neurons

**DOI:** 10.1038/srep44840

**Published:** 2017-03-22

**Authors:** Wenpo Chuang, Arun Sharma, Praveen Shukla, Guang Li, Moritz Mall, Kuppusamy Rajarajan, Oscar J. Abilez, Ryoko Hamaguchi, Joseph C. Wu, Marius Wernig, Sean M. Wu

**Affiliations:** 1 Stanford Cardiovascular Institute, Stanford University School of Medicine, Stanford, CA, USA; 2 Cardiovascular Center, Far Eastern Memorial Hospital, New Taipei City, Taiwan; 3 Institute for Stem Cell Biology and Regenerative Medicine, Stanford University School of Medicine, Stanford, CA, USA; 4 Department of Medicine, Division of Cardiology, Stanford University School of Medicine, Stanford, CA, USA; 5 Department of Biology, Stanford University School of Medicine, Stanford, CA, USA; 6 Department of Radiology, Molecular Imaging Program, Stanford University School of Medicine, Stanford, CA, USA; 7 Department of Pathology, Stanford University School of Medicine, Stanford, CA, USA.

## Abstract

Direct reprogramming of somatic cells has been demonstrated, however, it is unknown whether electrophysiologically-active somatic cells derived from separate germ layers can be interconverted. We demonstrate that partial direct reprogramming of mesoderm-derived cardiomyocytes into neurons is feasible, generating cells exhibiting structural and electrophysiological properties of both cardiomyocytes and neurons. Human and mouse pluripotent stem cell-derived CMs (PSC-CMs) were transduced with the neurogenic transcription factors *Brn2, Ascl1, Myt1l* and *NeuroD*. We found that CMs adopted neuronal morphologies as early as day 3 post-transduction while still retaining a CM gene expression profile. At week 1 post-transduction, we found that reprogrammed CMs expressed neuronal markers such as *Tuj1, Map2*, and *NCAM*. At week 3 post-transduction, mature neuronal markers such as *vGlut* and synapsin were observed. With single-cell qPCR, we temporally examined CM gene expression and observed increased expression of neuronal markers *Dcx, Map2*, and *Tubb3*. Patch-clamp analysis confirmed the neuron-like electrophysiological profile of reprogrammed CMs. This study demonstrates that PSC-CMs are amenable to partial neuronal conversion, yielding a population of cells exhibiting features of both neurons and CMs.

The use of direct reprogramming, or transcription factor overexpression to acquire an alternative cell fate, comprises a burgeoning area of study in regenerative medicine. The highly successful and reproducible reprogramming of fibroblasts into induced pluripotent stem cells (iPSCs) has paved the way for successes in direct reprogramming of fibroblasts into neurons and other somatic cell types^1^,^2^,^3^,^4^. However, iPSC production and downstream differentiation remains a laborious process, with the primary cell isolation, cellular reprogramming, and stem cell differentiation protocols taking upwards of a month and requiring multiple expensive reagents and often tedious, frequent maintenance. If the goal is to produce many cells for downstream purposes such as patient-specific *in vitro* disease modeling, pharmacological screening, or cell therapy, then direct reprogramming of one somatic cell type into another can offer major advantages of time and cost over iPSC-based reprogramming and differentiation. In additional contrast to iPSC-based methods, direct reprogramming lacks the creation of a pluripotent intermediate state, eliminating the possibility of teratoma formation during *in vivo* reprogramming. Current direct reprogramming protocols can produce a much smaller subset of somatic cell types than what is possible with pluripotent stem cell-based differentiation, but improvements in such protocols are rapidly underway^5^.

A variety of somatic cell types have been derived via direct reprogramming in recent years. Electrophysiologically-active neurons, oligodendroglial cells, and neural precursor cells can be generated from patient-specific fibroblasts with high efficiency, reducing the time, cost, and effort needed to generate patient specific iPSCs and differentiate them into neuronal cell types^1^,^6^,^7^. Notably, only a handful of defined neurogenic transcription factors, namely Brn2, Ascl1, Myt1l, and NeuroD (BAMN), are required for this process, which takes only a few days^8^. These neural cell types could be utilized to model neurological disorders such as Parkinson’s disease and Alzheimer’s disease, to screen for potential neurotoxicities associated with pharmacological compounds in active drug development, or to potentially treat neurodevelopmental diseases or acquired neurological disorders such as spinal cord injury-induced paralysis^9^. Neural cell types are not the only electrophysiologically-active somatic cell type that has been produced via direct reprogramming. Indeed, direct reprogramming of fibroblasts by overexpression of *Gata4, Mef2c, Tbx5, Hand2, Mesp1, Ets2*, Myocardin, or microRNAs has been reported to generate cells that exhibit some features of cardiomyocytes such as sarcomeric protein gene expression, striation patterns, and spontaneous calcium transients^10^,^11^,^12^,^13^,^14^. While it remains to be clarified whether any of these *in vitro* directly reprogrammed cardiac cells exhibit the full repertoire of gene expression and structural and biochemical function as their target cell (i.e. fully functional cardiomyocytes), this approach represents a major departure from the developmental paradigm of stem/progenitor cells giving rise to differentiated daughter cells. It raises the possibility that somatic cells may be converted to cardiovascular cells by transcription factor overexpression. As a testament to the rapid pace of this field, direct reprogramming has also been able to generate pancreatic beta cells from exocrine cells and, more recently, functional hepatocytes from fibroblasts^15^,^16^. A number of these directly-reprogrammed somatic cell types are currently being considered for clinical translation^17^.

The direct reprogramming protocols for the aforementioned somatic cell types will continue to improve over time. However, in the case of electrophysiologically active cell types such as cardiomyocytes and neurons, both cell types have currently been produced by reprogramming either dermal fibroblasts or cardiac fibroblasts, which are structurally simple and electrophysiologically inert. To further evaluate the strength and efficacy of the direct reprogramming process, specialized, electrophysiologically-active cell types derived from different germ layers should also be tested for their propensity to interconvert. As a proof-of-principle, we examined the ability of recently described neurogenic reprogramming factors *Brn2, Ascl1, Myt1l* (BAM) (for mouse), plus *NeuroD* (BAMN) (for human) to convert mouse and human pluripotent stem cell-derived cardiomyocytes (PSC-CMs) into induced neurons^2^. Although the mesoderm-derived cardiac cell types and ectoderm-derived neurons arise from separate developmental origins, specialized cardiomyocytes of the cardiac electrical conduction network, such as Purkinje fibers, overlap with neurons in terms of gene expression for calcium and potassium channels needed for action potential propagation, intermediate filaments for the maintenance of spiny structure, and neural crest-associated markers^18^,^19^,^20^. These similarities may facilitate the reprogramming process between the two electrophysiologically active cell types.

This work provides novel insight into direct somatic cell reprogramming by testing the strength of the neurogenic BAMN factors in activating the neurodevelopmental program in a non-ectodermal, highly-specialized, electrophysiologically active cardiac cell type, namely cardiomyocytes. We utilized single-cell qRT-PCR, immunofluorescence, time-lapse microscopy, and patch-clamp electrophysiology to characterize the sequential process of human and mouse PSC-CM neuronal conversion. We also identified partially reprogrammed, neuron-cardiomyocyte cells that harbor both cardiomyocyte and neuronal gene expression.

## Results

### Induction of Neuronal Gene Expression in Mouse Embryonic Stem Cell-Derived Cardiomyocytes

**Figure 1:**
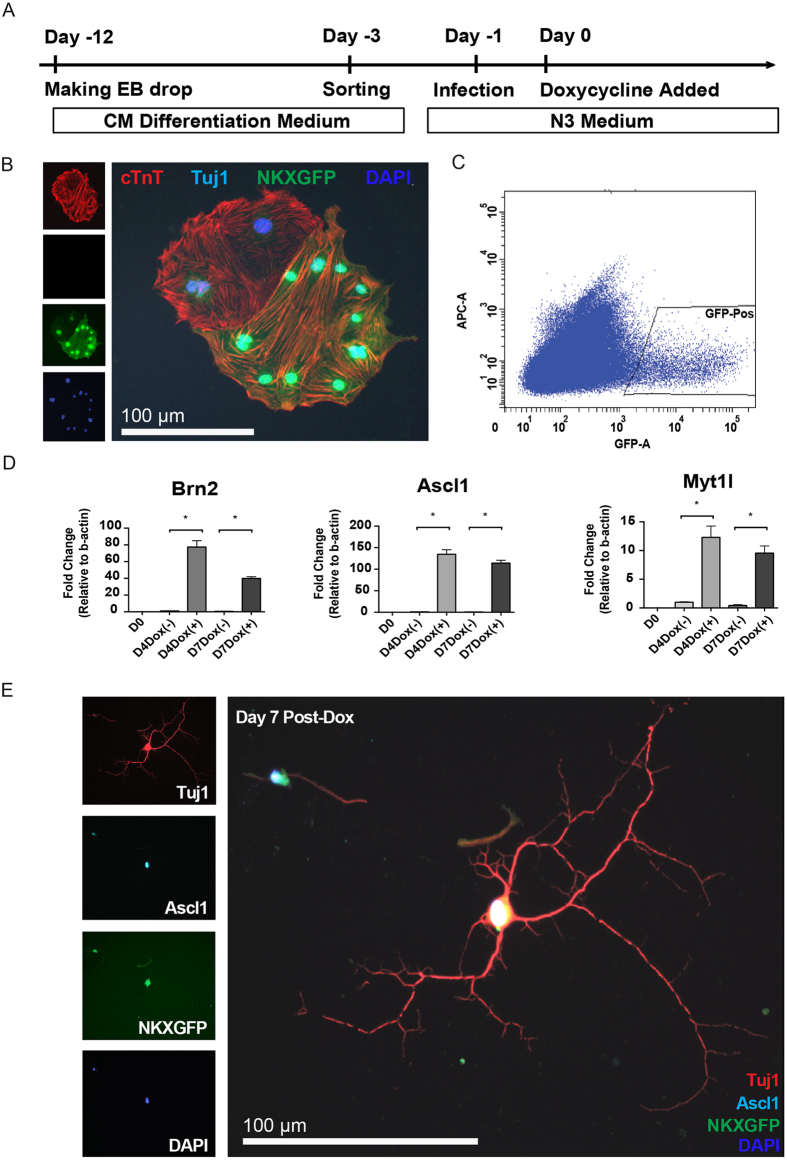
Induction of Neuronal Gene Expression in Mouse Embryonic Stem Cell-Derived Cardiomyocytes. (**A**) Timeline of mESC differentiation into CMs and lentiviral transduction of FACS-purified CMs. Nkx2-5-eGFP^+^ mESCs are differentiated as EBs for 9 days and eGFP^+^ CMs are isolated by FACS and transduced with lentivirus containing BAM and subsequently cultured in N3 medium. Dox was added 24 hours after lentiviral transduction to activate BAM expression. (**B**) Immunofluorescence images of eGFP^+^ mESC-CMs prior to lentiviral transduction demonstrate the expression of typical sarcomeric proteins such as cardiac troponin T (*cTnT*) but absence of neuronal proteins such as tubulin J (*Tuj1*). (**C**) Flow cytometry plot showing the gating used to purify Nkx2-5-eGFP^+^ CMs. (**D**) qRT-PCR for the expression of BAM factors at days 4 and 7 days after Dox administration. *Indicates *P* < 0.05. N = 3 biological replicates. Data are expressed as means ± SEM. (**E**) Immunofluorescent staining analysis of neuronal marker expression in reprogrammed Nkx2-5-eGFP^+^ mESC-CMs. Note the expression of neuronal proteins such as Tuj1 and Ascl1 along with Nkx2-5-eGFP^+^.

The Nkx2-5 cardiac enhancer and base promoter-eGFP (Nkx2-5-eGFP^+^) mouse embryonic stem cells (mESCs) were differentiated as hanging drop embryoid bodies (EBs) for 9 days into eGFP+ CMs (Fig. 1A)^21^. Prior to transduction with Doxycycline (Dox)-inducible lentiviruses expressing BAM, these eGFP+ CMs show prominent expression of sarcomeric proteins such as cardiac troponin T (cTnT) but not the neuronal marker neuronal specific class III beta-tubulin (Tuj1) (Fig. 1B). eGFP+ CMs were then purified by fluorescence activated cell sorting (FACS) (Fig. 1C) and transduced with Dox-inducible lentiviruses expressing BAM. Following transduction and treatment with Dox, the Dox-treated mouse embryonic stem cell-derived cardiomyocytes (mESC-CMs) showed elevated expression of BAM at days 4 and 7 post-transduction by 12- to 120-fold, respectively, over cells without Dox treatment (Fig. 1D). Interestingly, cells with spiny neuronal projections, including dendrite-like processes, and Tuj1 expression were observed in transduced mESC-CMs at 7 days post Dox treatment (Fig. 1E).

### Characterization of Sequential Conversion into Neuron-Cardiomyocyte Cells by BAM Lentivirus-Transduced mESC-CM

**Figure 2:**
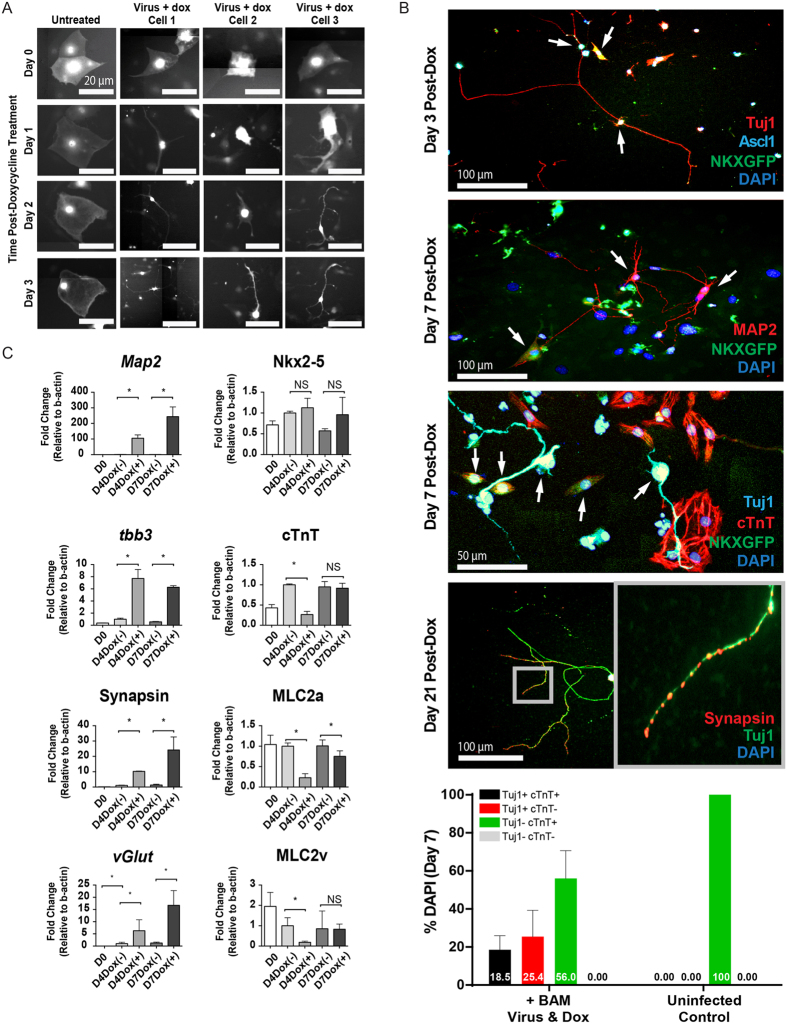
Characterization of Sequential Conversion into Neuronal-like Cells by BAM Lentivirus-Transduced mESC-CMs. (**A**) Still frames from time-lapse microscopy recordings illustrate morphological transformation of individual eGFP+ mESC-CMs 3 days following transduction with neurogenic lentiviruses and addition of Dox. (**B**) Immunofluorescent staining analysis of neuronal marker expression in reprogrammed mESC-CMs at three distinct time points after Dox treatment. Note that the expression of *Ascl1* and *Tuj1* appears within 3 days of Dox treatment (top). After 7 days of Dox treatment, some cells expressed both *Tuj1* and *Map2*, and display axon-like projections (middle panels). Some cells also co-express cardiac marker Nkx2-5 and neuronal marker Tuj1. After 21 days of Dox treatment, transduced cells demonstrate the phenotype of a remarkably mature neuronal cell with elongated, axon-like projections and expression of mature markers such as synapsin (lower panel). Partially-converted cells are indicated by arrows. At day 7, ~18.5% of transduced mouse PSC-CMs express both the neuronal marker *Tuj1* and cardiomyocyte marker *cTnT* within 1 week of Dox addition while 25.4% of cells are *Tuj1*+*cTnT*−. N = 66 cells counted in total. (**C**) qRT-PCR results confirm up-regulation of neuronal markers (left column) up to 7 days following transduction with neurogenic lentivirus and addition of Dox. We also examined cardiac-specific markers (right column). Notably, the expression of cardiac-specific protein Nkx2-5 is largely retained following transduction. We observed a drop in the expression of atrial specific marker MLC2a at both day 3 and day 7. We also observed a drop in ventricular-specific marker MLC2v and cardiac troponin T (cTnT) at day 4, but this returned the same levels as the dox-untreated cells by day 7. *Indicates *P* < 0.05, NS indicates not significant. N = 3 biological replicates. Data are expressed as means ± SEM.

To document the step-wise transition of Nkx2-5-eGFP^+^ mESC-CMs into neuron-like cells, we performed time-lapse microscopy of BAM lentivirus-transduced eGFP+ CMs for three days following Dox treatment (Fig. 2A, Supplemental Movie 1). Several eGFP+ cells readily underwent morphological change after BAM overexpression for only 3 days and displayed dendrite-like projections with an elongated shape. These cells expressed neuronal markers such as *Tuj1* (Fig. 2B). With a longer treatment period, additional neuronal markers such as *Map2* can be visualized. Some cells exhibited simultaneous expression of cardiac marker Nkx2-5 and of neuronal markers. Aside from the presence of these neuronal genes in cells that were previously CMs, some of these cells also expressed synapsin, a mature neuronal marker, in the expected distribution at nerve terminals at 21 days after Dox treatment. We determined that approximately 25% of mouse PSC-cardiomyocytes initiated neuronal conversion by their *Tuj1*+*cTnT*− expression, whereas approximately 18% of cells exhibited a partial neuronal phenotype by their dual *Tuj1*+*cTnT*+ cardiomyocyte-neuron gene expression. Real-time quantitative PCR analysis on mouse CMs treated with BAM lentivirus transduction and Dox induction confirmed the increased expression of neuronal specific genes (Fig. 2C, left column). Expression of cardiac marker Nkx2-5 remained largely unchanged during the conversion process. Cardiac-specific marker cTnT decreased transiently in doxycycline-treated transduced cells at day 4 post-dox treatment. Expression of atrial specific marker MLC2a was reduced in doxycycline-treated transduced cells at days 4 and 7 post-Dox. Notably, expression of most, if not all, sarcomeric proteins were only transiently reduced. This is likely due to the attenuation of BAMN expression at day 7 resulting in failure of maintenance of neuronal reprogrammed phenotype in many cells (see Fig. 1D) and the re-expression of cardiac markers. These results reflect the heterogeneity of the conversion process and the presence of intermediate, partially-converted, cardiomyocyte-neuron cells. (Fig. 2C, right column).

### Neuronal Conversion of Human iPSC-Derived CMs Using BAMN-expressing Lentiviruses

**Figure 3:**
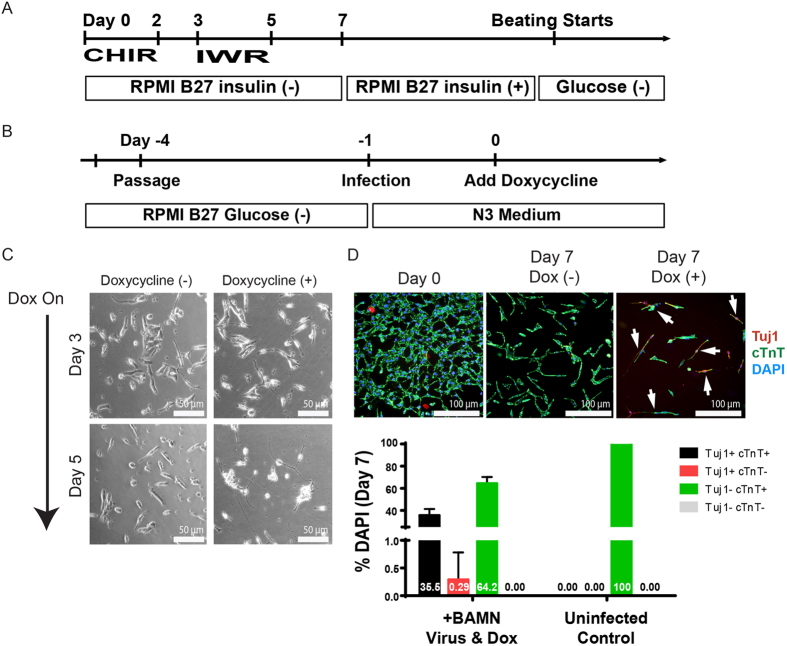
Neuronal Conversion of Human iPSC-Derived CMs Using BAMN-expressing Lentiviruses. (**A**) Experimental protocol for the generation of hiPSC-CMs and their purification via glucose starvation. (**B**) Experimental protocol for the transduction of purified human CMs with BAMN containing lentiviruses and their subsequent culturing. (**C**) Bright-field microscopy of transduced hiPSC-CMs exhibiting progressively more neuronal morphologies from three to five days after Dox treatment. (**D**) Immunofluorescent images and quantitative analysis of neuronal and cardiac gene expression at 7 days after induction of BAMN expression. Note that ~35.5% of transduced hiPSC-CMs express both the neuronal marker *Tuj1* and cardiomyocyte marker *cTnT* within 1 week of Dox addition while only 0.29% of *Tuj1*+ cells lose their *cTnT* expression. Cardiomyocyte-neuron cells are indicated by arrows. N = 2414 cells counted in total.

To determine whether the neuronal conversion of CMs by BAM factor overexpression is conserved across different species, we differentiated human induced pluripotent stem cells (hiPSCs) into cardiomyocytes (CMs) using a previously-published protocol^22^ (Fig. 3A) and treated these purified hiPSC-CMs with lentiviruses expressing BAMN, which has been shown to induce neuronal reprogramming in human cells (Fig. 3B)^23^. Following lentiviral transduction and Dox treatment, a phenotypic conversion of CM towards neuronal morphologies was observed as early as 3 days and became progressively more prominent by 5 days post-Dox treatment (Fig. 3C). Similarly to mESC-CMs, transduced hiPSC-CMs expressed human neuronal marker Tuj1 in conjunction with the pan-cardiomyocyte marker cTnT after 7 days of Dox treatment (Fig. 3D). Quantification of transduced cell populations (as determined by their ectopic expression of *Ascl1*) indicates that approximately 35% of transduced human iPSC-CMs adopted a *Tuj1*+*cTnT*+ phenotype, supporting the initiation of their cardiomyocyte-to-neuron cell transition process. However, only 0.29% of transduced human CMs adopted a more neuronal phenotype by their *Tuj1*+*cTnT*− expression profile. Single-cell quantitative RT-PCR was also performed on BAMN-treated hiPSC-CMs to assess the changes in neuronal and cardiac gene expression in the same cell over the three-week timecourse (Supplemental Figure 1). At an early timepoint (1 week), purified single hiPSC-CMs adopted a gene expression profile similar to that of control primary human heart tissue, where the expression of *cTnT* is high and the expression of neuronal genes such as *Map2* is absent. At a later timepoint (2 weeks), a change in gene expression pattern is observed where the neuronal gene expression is increased in many cells while their cardiomyocyte gene expression is either unchanged or decreased. In one cell (2 WK—17), the expression of cardiomyocyte genes (*MYH6, TNNT2*) was completely silenced while the expression of all three neuronal genes (*DCX, MAP2, TBB3*) were present.

### Electrophysiological Characterization of Neuronal-like Cells Derived from Human iPSC-Derived CMs

**Figure 4:**
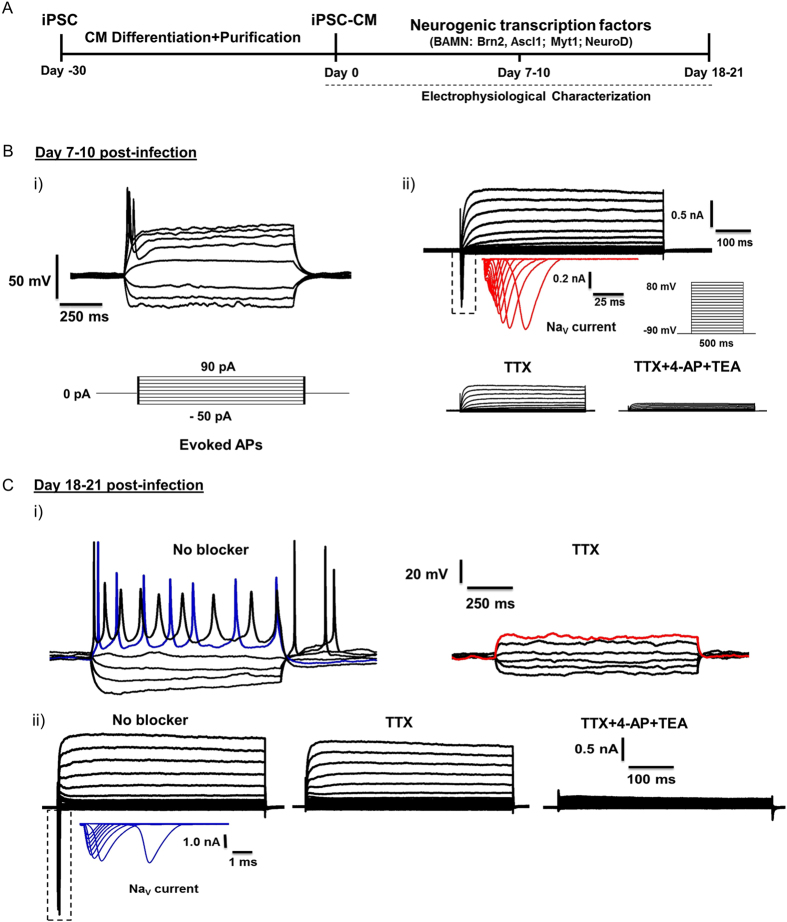
Electrophysiological Characterization of Neuronal-like Cells Derived from Human iPSC-Derived CMs. (**A**) Schematic representation of experimental design for inducing neuronal phenotype in hiPSC-derived cardiomyocytes transduced with neurogenic transcription factors (BAMN: Brn2, Ascl1; Myt1; NeuroD). (**B**) (i) Abortive action potential (AP) generation in a cell exhibiting the neuronal morphology at day 7–10 post transduction with BAMN factors with step-current injection. N = 12 cells recorded. Representative trace shown. (ii) Sample traces of voltage gated Na^+^ (inset in red = magnified view of the boxed area) and K^+^ currents recorded from a cell exhibiting neuronal morphology at day 7–10 post transduction with BAMN factors. Cells were held at −90 mV; step depolarization from –90 mV to +80 mV at 10 mV intervals for 50 ms was delivered. N = 6 cells recorded. Representative trace shown. (**C**) (i) Repetitive action potentials (AP) generation in cells exhibiting the neuronal morphology at day 18–21 post transduction with BAMN factor with step-current injection and were completely blocked by TTX. N = 15 cells recorded. Representative traces shown. (ii) Representative traces of whole-cell currents in voltage-clamp mode in cells exhibiting neuronal morphology at day 18–21 post transduction with BAMN factors (left panel). The inward Na^+^ currents (inset in red or blue = magnified view of the boxed area) was observed and could be blocked by tetrodotoxin (TTX) (middle panel), and an outward K^+^ current could be blocked by tetraethylammonium (TEA) +4-aminopyridine (4-AP). N = 7 cells recorded. Representative traces shown.

At baseline, we determined that the day 30 hiPSC-CMs we employed to initiate the neuronal reprogramming process were of atrial-like, ventricular, like, and nodal-like subtypes without any neuronal features (Supplemental Figure 2, Supplemental Tables 1 and 2). To determine whether the cells that exhibit neuronal morphologies from reprogrammed hiPSC-CMs also display electrophysiological characteristics of functional neurons, we performed single-cell current-clamp recordings in reprogrammed hiPSC-CMs at week 1 (7–10 days) and week 3 (18–21 days) after transduction with BAMN lentivirus and Dox treatment (Fig. 4A). We injected a 30 pA depolarization current of 1 s duration to test the firing ability of these cells, since the ability to fire a succession of repetitive action-potentials (AP) upon membrane depolarization is a unique characteristic of mature neurons^24^. At week 1 after transduction, the neuronal-shaped cells derived from hiPSC-CMs fired only abortive APs (Fig. 4Bi). Interestingly, at week 3 (i.e. day 21) of transduction, the neuronal-shaped cells could fire multiple and repetitive APs (Fig. 4Ci). These week 3 APs were similar to those exhibited by primary rat neuronal cultures (Supplemental Figure 3). There appears to be a progressive increase in the Na_v_ and K_v_ currents from days 7 to 21 as well which correlates well with the increased ability of neuronal-shaped cells to fire repetitive APs. Since the amplitude of APs and membrane excitability is determined by the sodium (Na_v_) and potassium (K_v_) currents, we characterized the changes of Na_v_ and K_v_ currents at week 1 and week 3 post-transduction. In whole-cell voltage-clamp mode, we observed rapidly inactivating inward Na_v_ currents and persistent outward K_v_ currents in response to depolarizing voltage steps that could be blocked by tetrodotoxin (TTX) and a cocktail of 4-aminopyridine (4-AP) and tetraethylammonium (TEA), respectively (Fig. 4Cii). Taken together, these data show for the first time the feasibility of inducing neuronal electrophysiological phenotype in human iPSC-CMs by transduction with neurogenic transcription factors.

## Discussion

Using lentiviruses that overexpress neurogenic transcription factors previously shown to induce neuronal conversion from fibroblasts, we found that mouse and human PSC-CMs are amenable to partial neuronal phenotype conversion. We observed a significant number of cells expressing markers of both cardiomyocytes and neurons, suggesting the presence of cells in an intermediate, partially-reprogrammed state. Electrophysiological studies of reprogrammed cells with neuronal morphologies demonstrated the presence of mature neuronal action potentials in some of the reprogrammed cells. Taken together, these results demonstrate the ability of neuronal transcription factors to directly reprogram mesoderm-derived PSC-CMs into electrophysiologically active cells that exhibit variable degrees of neuron phenotype.

Advances in lineage-specific reprogramming by transcription factor overexpression have raised significant promise that this approach may be useful to generate various types of cells for cell-based therapy. The most successful example of this approach is the generation of PSCs from fibroblasts by overexpression of highly potent transcription factors such as Oct4 and Sox2 that has been shown to be able to erase fibroblast epigenetic markings and induce epigenetic markings of pluripotent stem cells^25^,^26^. Further progress in this realm of research has now led to reprogramming of fibroblasts into various cell lineages such as neurons, cardiomyocytes, blood cells, hepatocytes, and oligodendrocytes with variable efficiency. Our results demonstrate that neuronal transcription factors can partially reprogram PSC-CMs into neuron-like cells. These results support the presence of pioneer transcription factor activity in BAM/BAMN for the neuronal lineage, given their potency and efficiency to induce neuronal lineage conversion regardless of the differences in germ layer origin or structural specialization of the starting cell population. Indeed, a very recent study employing chromatin immunoprecipitation of BAM factors followed by genome-wide high-throughput sequencing has confirmed that Ascl1 most likely plays a pioneer factor role during neuronal reprogramming^8^. This factor may open closed chromatin and allow for the entry of other neuronal transcription factors, further driving the neuronal conversion process by silencing non-neuronal genes. Regarding the silencing of the cardiogenic program, a recent study addresses the intermediate reprogrammed cell phenotype using single cell RNA sequencing and found that a competing myogenic transcriptional program must be silenced in order to complete the neuronal reprogramming process^27^.

Our data demonstrate that many PSC-CMs are partially reprogrammed as demonstrated by their expression of both CM and neuronal genes (Figs 2 and 3D, Supplemental Figure 1). For example, several reprogrammed cells are positive for the early cardiac marker Nkx2-5 as well as for neuronal markers such as Tuj1, Ascl1, and MAP2 (Fig. 2B). As mentioned by studies looking at cardiomyocyte or neuron production through direct reprogramming, there are a number of intermediate and partially-reprogrammed cells produced during the direct conversion process from fibroblast to cardiomyocyte and from fibroblast to neuron^1^,^10^. Interestingly, we observed a higher efficiency of neuronal conversion in mouse cardiomyocytes in comparison to human cardiomyocytes. The fact that the human PSC-CMs in our study achieved *Tuj1*+*cTnT*− neuronal conversion at an extremely low rate (0.29%) reflects the difficulty of complete conversion from cardiomyocytes to neurons in this population.

The initial study that used BAM to produce induced neurons demonstrated that most of the converted cells were excitatory, glutamatergic neurons, largely of the cortical variety. That study also found some induced neurons expressed markers of GABAergic neurons^1^. However, despite the presence of some features of mature neurons and spontaneous action potentials in our reprogrammed cells that resemble the action potential of fully mature neurons, many cells continue to express cardiomyocyte genes. This is reminiscent of the immature cells produced in previous studies of *in vitro* direct reprogramming of fibroblasts into CMs^10^,^28^,^29^. Given the immaturity of these cardiomyocyte-converted neuron-like cells, would they be more amenable to conversion *back* to the cardiomyocyte state as well? Previous studies have indeed demonstrated that somatic tissues, namely fibroblasts, can be directly converted into cardiomyocytes, in both humans and mice, using the defined transcription factors Gata4, Mef2c, and Tbx5 (GMT)^10^,^30^. However, we have previously demonstrated that this conversion process is extremely inefficient, with less than 5% cardiomyocyte-like cells being produced from this fibroblast conversion process^28^. As such, we have not attempted to use the GMT protocol to convert cells such as the CM-neuronal cells back into cardiomyocytes. The electrophysiological, structural, and functional immaturity of directly reprogrammed cells remains a major issue in stem cell biology and regenerative medicine applications^31^. Further work will be needed to improve the fidelity of the reprogrammed phenotype and the efficiency of lineage conversion to be able to achieve a homogenous population of the desired cell type.

In summary, we have, for the first time, converted mouse and human CMs into neuronal-like cells. Many of the converted cells exhibit partial features of both CMs and neurons. The fact that only three or four neuronal transcriptional factors are needed to convert mesoderm-derived, electrophysiologically-active CMs into ectoderm-derived, electrophysiologically-active neuronal-like cells suggests that distinct cell fates are regulated by very few key transcription factors.

## Methods

All experiments were performed in accordance with relevant guidelines and regulations of the Stanford University School of Medicine.

### *In vitro* differentiation of murine Nkx2-5-eGFP ESCs and flow sorting for eGFP^+^ ESC-CMs

Mouse ESCs were cultured in accordance with previous studies and adapted to gelatin-coated dishes in the presence of leukemia inhibitory factor (LIF) for 2 days prior to differentiation^32^. CM differentiation of ESCs was performed per a previously-published protocol^21^. On the day of flow sorting, embryonic bodies (EBs) were digested with trypsin/EDTA for 3 minutes followed by collagenase A + B solution (10 mg/ml each) for 30 minutes. These cells were then re-suspended in HBSS (Life Technologies) with 20% FCS (Sigma). The eGFP-expressing cells were analyzed and sorted using a FACS Aria II system, and flow cytometry data were analyzed using FACS Diva Software (Becton Dickinson).

### Maintenance of hiPSCs and differentiation of hiPSCs to hiPSC-CMs

Human iPSCs were maintained on Matrigel (BD Biosciences) coated plates in E8 medium (Life Technologies), which was changed daily^33^. Prior to cardiac differentiation, hiPSCs were passaged once every four days with 0.5 mM EDTA at 37 °C for 5 minutes. After hiPSCs had achieved 95% confluence, cardiac differentiation was initiated by treating cells with 6 μM CHIR99021 (Selleck Chemicals) in RPMI + B27 without insulin for 48 hours (day 0 to day 2) according to a previously-published protocol^23^. The medium was then changed to RPMI + B27 without insulin for 1 day, and 5 μM IWR1 (Selleck Chemicals) was added between days 3 to 5 of differentiation. From day 5 to 7, cells were returned to RPMI + B27 without insulin and without small molecules. Cells were then maintained on RPMI + B27 with insulin from day 7 onwards. Once spontaneous beating could be observed (usually between days 10 to 12 of differentiation), the medium was changed to RPMI without glucose + B27 with insulin for glucose starvation for 3 days to eliminate non-CMs in culture. This step typically resulted in greater than 90% CM purity. The surviving CMs were then passaged with trypLE^TM^ (Life Technologies) and replated for further studies.

### Viral transduction

Replication-incompetent, VSVg-coated lentiviral particles carrying doxycycline (Dox)-inducible cDNA for BAM (mouse studies) or BAMN (human studies) expression were packaged in 293 T cells with a Fugene HD kit, where the DNA to Fugene volume ratio of 1 to 2.5 was given per the manufacturer’s suggested protocol. Viruses produced were further concentrated by PEG-it virus concentration solution (System Biosciences) and stored at −80 °C until needed. To induce neuronal phenotype conversion in mouse and human PSC-CMs, we transduced FACS-purified eGFP+ CMs from *in vitro* differentiated Nkx2-5-eGFP ES cells and human iPSC-CMs at 2 passages after glucose-starvation with prepared lentiviruses according to previously published protocols^1^,^2^. After PSC-CMs had been incubated for 16–20 hours in lentivirus-containing media, the cells were switched into fresh N3 media containing doxycycline (2 μg/ml) to activate BAM or BAMN expression. The media was changed every 2–3 days for the remainder of the culture period.

### Immunofluorescence

For immunostaining, cultured cells were fixed with 4% paraformaldehyde for 20 minutes at room temperature and then washed with PBS. Cells were then permeabilized with Triton X-100 and blocked with 1% BSA and goat serum. The following antibodies were used for immunofluorescence: rabbit anti-tubulin J1 (*Tuj1*) (Covance, 1:1000); mouse anti-*Tuj1* (Covance, 1:1000); mouse anti-microtubule associated protein 2 (*Map2*) (Sigma M4403); E028 rabbit anti-synapsin (a kind gift from Dr. Thomas Südhof at Stanford University, 1:500).

### Electrophysiology

For electrophysiological assays, cultured hiPSC-CMs or reprogrammed cells were dissociated using TrypLE for 10 min at 37 °C, centrifuged at 200xg, suspended in RPMI media supplemented with B27 + insulin (RPMI + B27 with insulin), filtered through a 70 or 100 μM cell strainer (BD Biosciences), and plated as single cells (1 × 10^5^ cells per well of a 24-well plate) on No. 1 (8 mm) glass cover slips (Warner Instruments, Hamden, CT, USA) coated with Matrigel (1:50 ratio). Immediately after plating, cells were grown in RPMI + B27 with insulin media supplemented with 2 μM thiazovivin and allowed to attach for 48–72 hours, changing the media every other day. Cells were analyzed at indicated times after transduction. Using an EPC-10 patch-clamp amplifier (HEKA, Lambrecht, Germany), whole-cell action potentials and currents were recorded in standard current and voltage clamp mode, respectively, as shown previously^34^,^35^,^36^. Glass micropipettes (2–3 MΩ tip resistance) were fabricated from standard wall borosilicate glass capillary tubes (Sutter BF 100–50^−10^, Sutter Instruments, Novato, CA) using a programmable puller (P-97; Sutter Instruments) and filled with the following intracellular solution: 123 mM K-gluconate, 10 mM KCl, 1.0 mM MgCl_2_, 10 mM HEPES, 1 mM EGTA, 0.1 mM CaCl_2_, 1 mM K_2_ATP, 0.2 mM Na_4_GTP and 4 mM glucose; pH was adjusted to 7.2 with KOH. Membrane potentials were kept around −65 to −70 mV, and step currents were injected to elicit action potentials. For voltage-dependent current recordings, the same internal solution as was used. Whole-cell sodium and potassium currents were recorded at a holding potential of −90 mV, and 200 ms voltage steps ranging from −80 mV to +90 mV were delivered at 10 mV increments. Series resistance and cell capacitance were compensated to between 50–80% in all voltage clamp recordings as shown previously^35^,^36^. All recordings were made at room temperature except for the cardiac action potentials, which were recorded at 35–37 °C. The extracellular solution contained 140 mM NaCl, 5 mM KCl, 2 mM CaCl_2_, 2 mM MgCl_2_, 10 mM HEPES, and 10 mM glucose; pH was adjusted to 7.4 with NaOH. A single beating CM or cell exhibiting neuronal morphology was selected for action potentials (APs) or current recording. Data were acquired using PatchMaster software (HEKA, Germany), digitized at 1.0–10 kHz and analyzed using IGOR Pro V:6.2.2.2, a custom-built MatLab script, and MS Excel software. The following are the criteria used for classifying observed APs into ventricular-, atrial- and nodal-like hiPSC-CMs: for ventricular-like cells, the criteria were a negative maximum diastolic membrane potential (less than or equal to 50 mV), a rapid AP upstroke, a long plateau phase, AP amplitude >90 mV and AP duration at 90% repolarization/AP duration at 50% repolarization (APD_90_/APD_50_) <1.4. For atrial-like cells, the criteria were an absence of a prominent plateau phase, a negative diastolic membrane potential (less than or equal to 50 mV) and APD_90_/APD_50_ > 1.7. For nodal-like cells, the criteria were a more positive MDP, a slower AP upstroke, a prominent phase 4 depolarization and APD_90_/APD_50_ between 1.4 and 1.7^34^.

### Gene expression analysis

For gene expression analysis, cultured cells were rinsed with PBS, immediately harvested using Trizol^®^ reagent (Invitrogen, Carlsbad CA), and stored at −80 °C until needed. Total RNA from each sample was purified from cell lysate using QIAGEN RNAeasy kit per the manufacturer’s instructions. Quantitative PCR was performed on cDNA made from reverse-transcribed RNA using the iScript^TM^ cDNA synthesis kit (BioRad, Hercules, CA). For single cell analysis, cells were rinsed with PBS and then digested with a combination of trypsin and collagenase A and B (10 mg/mL each). Single cells were then manually selected under bright-field microscopy and subjected to cell lysis and target gene-specific reverse transcription and amplification according to a previously-published protocol^37^. Quantitative PCR was then performed using the BioRad CFX96TM qPCR system with SYBR^®^ Green substrate (BioRad, Hercules, CA) for 40 cycles. A gene expression heat map for single cell analysis was generated in R software.

### Statistical analysis

Data presented as mean ± standard error of the mean (SEM) unless otherwise specified. Comparisons were conducted via Student’s t-test with significant differences (*) defined by P < 0.05.

## Additional Information

**How to cite this article:** Chuang, W. *et al*. Partial Reprogramming of Pluripotent Stem Cell-Derived Cardiomyocytes into Neurons. *Sci. Rep.* **7**, 44840; doi: 10.1038/srep44840 (2017).

**Publisher's note:** Springer Nature remains neutral with regard to jurisdictional claims in published maps and institutional affiliations.

## Supplementary Material

Supplemental Movie 1

Supplementary Information
