# Supplementary material for: Partial Reprogramming of Pluripotent Stem Cell-Derived Cardiomyocytes into Neurons
Source: Sci Rep. 2017 Mar 22;7:44840. doi: 10.1038/srep44840 (PMC5361100; doi:10.1038/srep44840)
Supplement: Supplementary Information [file srep44840-s2.pdf]

SUPPLEMENTAL INFORMATION

Partial Reprogramming of Pluripotent Stem Cell-Derived Cardiomyocytes into Neurons

Wenpo Chuang[1, 2+], Arun Sharma[1, 3+], Praveen Shukla[1, 3+], Guang Li[1, 3], Moritz Mall[3], Kuppusamy Rajarajan[1, 3], Oscar J. Abilez[1, 4], Ryoko Hamaguchi[1, 5], Joseph C. Wu[1, 3, 4, 6], Marius Wernig[3, 7], Sean M. Wu[1, 3, 4]*

Affiliations:
[1]Stanford Cardiovascular Institute, Stanford University School of Medicine, Stanford, CA
[2]Cardiovascular Center, Far Eastern Memorial Hospital, New Taipei City, Taiwan
[3]Institute for Stem Cell Biology and Regenerative Medicine; [4]Department of Medicine, Division of Cardiology; [5]Department of Biology; [6]Department of Radiology, Molecular Imaging Program; and [7]Department of Pathology, Stanford University School of Medicine, Stanford, CA

Brief Title: Neuronal Reprogramming of Cardiomyocytes

Body Word Count: 4500

Keywords: Cardiomyocyte, stem cell, arrhythmia, neuron

+Indicates equal contribution

***Correspondence:**
Sean M. Wu, MD, PhD
Lorry I. Lokey Stem Cell Research Building, Rm G1120A
265 Campus Drive
Stanford, CA 94305-5454
(650)724-4498 (office)
(650)724-4689 (fax)
E-mail: smwu@stanford.edu

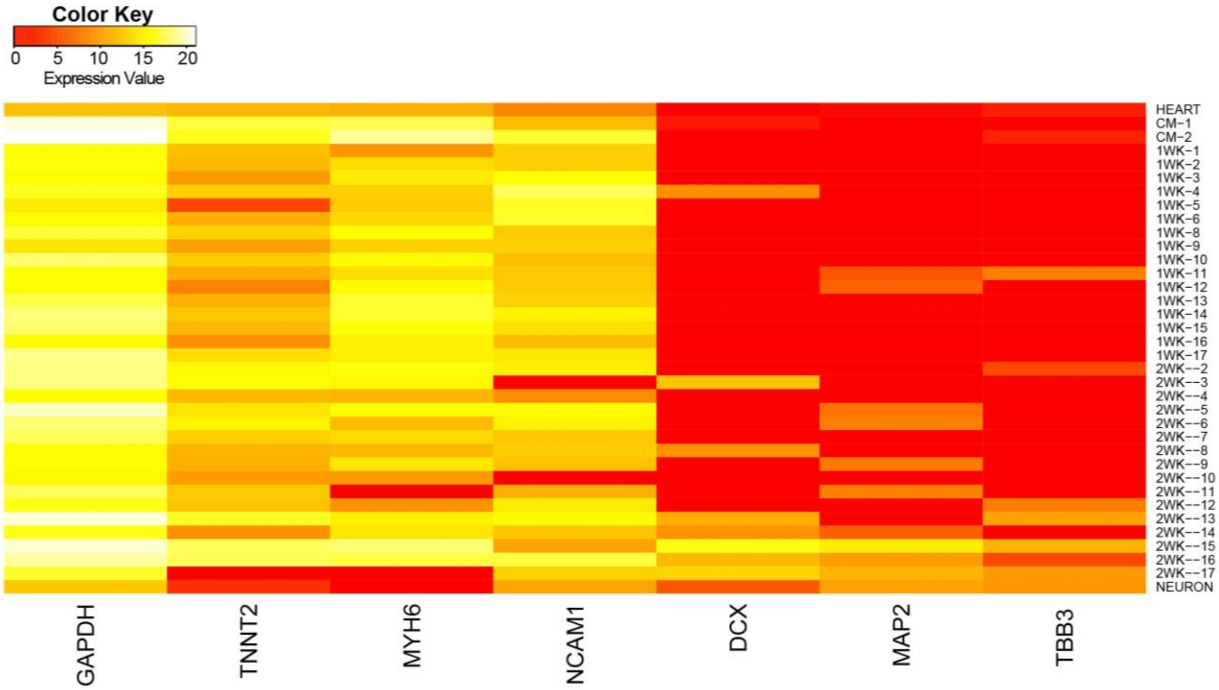

Supplemental Figure 1: Analysis of human iPSC-CM conversion into induced neurons by single cell transcriptional profiling. Human iPSC-CM were transduced with BAMN expression lentiviruses and treated with doxycycline to induce BAMN overexpression for 1 and 2 weeks. At the indicated time point, single cells were manually selected and lysed and qRT-PCR was performed against the indicated cardiac (TNNT2, MYH6, NCAM1) and neuronal (DCX, MAP2, TBB3) genes as well as a house keeping gene (GADPH). Each row represents one cell. HEART and NEURON represents control RNA from commercial heart and neuron tissues. CM represents control RNA from hiPSC-CM.

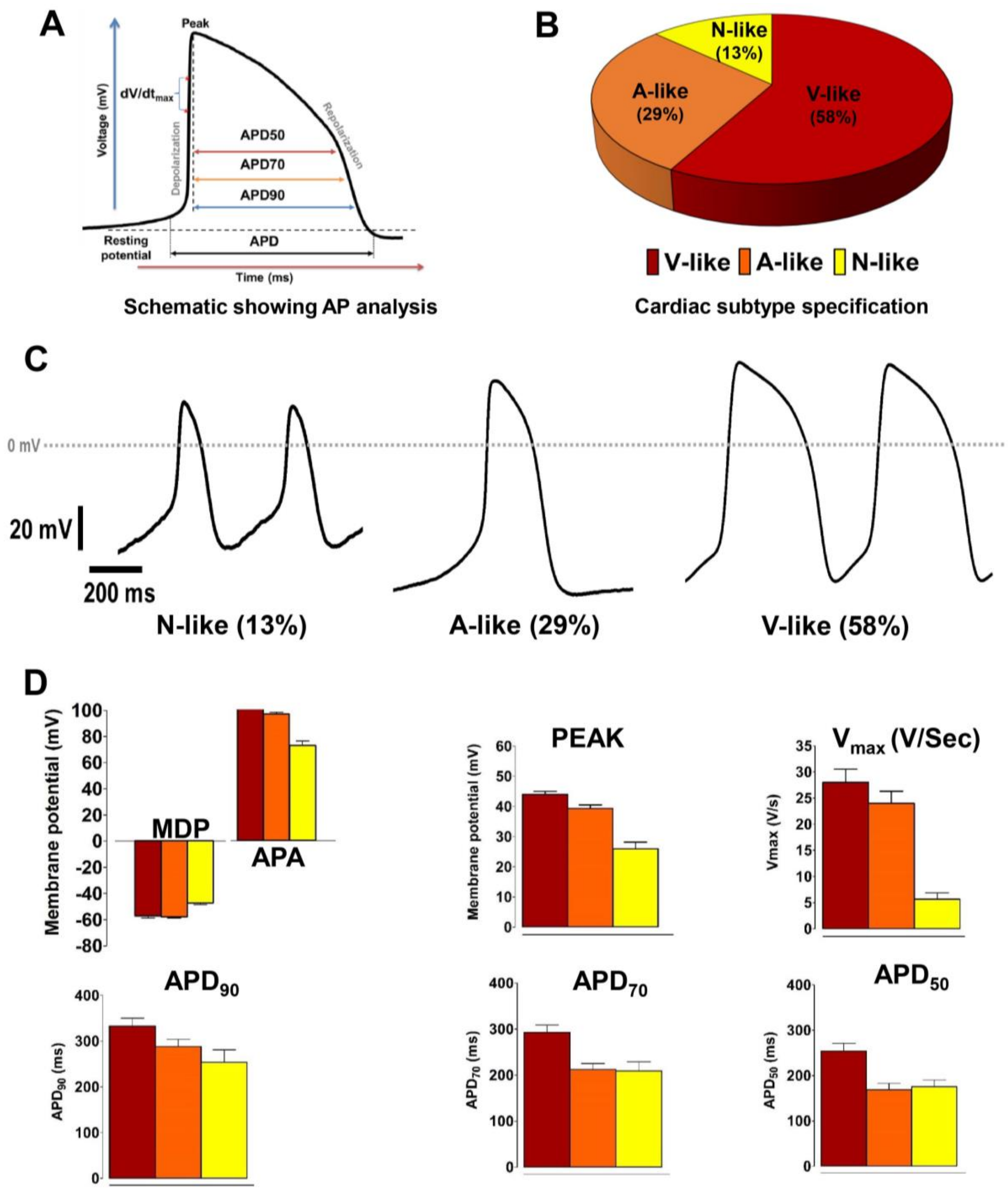

Supplemental Figure 2: Electrophysiological characterization of hiPSC-cardiomyocytes (hiPSC-CMs) at day 30-32 day of differentiation. (A) Schematic diagram of an AP trace, showing how results were analyzed to calculate action potential duration at 50, 70, and 90% repolarization (APD_{50}, APD_{70}, and APD_{90}, respectively). (B) Subtype distribution of hiPSC-CMs, (*n* = 24). (C) Representative action potential (AP) recordings using whole cell patch clamp of three major CM subtypes. Cells exhibit AP morphologies that can be categorized as ventricular (V)-, atrial (A) - or nodal (N) -like CMs. (D) Patch clamp recordings of hiPSC-CMs, demonstrating maximal diastolic potential (MDP), action potential amplitude (APA), overshoot/peak voltage, V_{max} (maximal rate of depolarization), APD_{50}, APD_{70}, and APD_{90}. Statistical information in Supplemental Table 2.

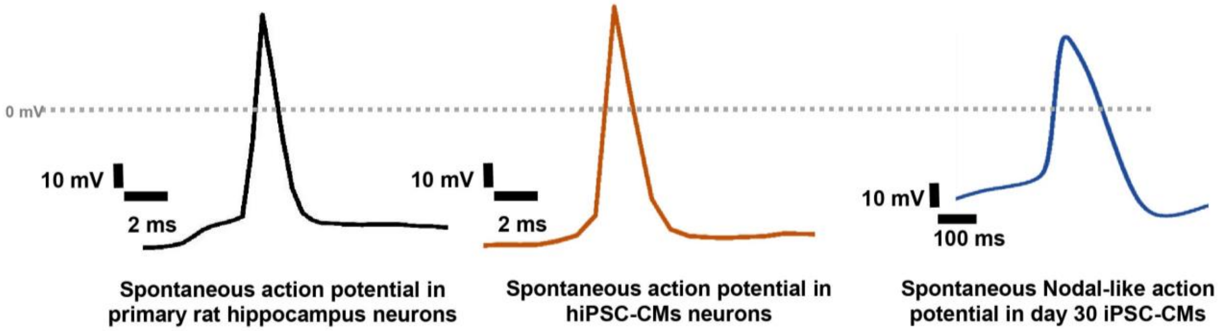

Supplemental Figure 3: A comparative summary of the action potential (AP) waveform recorded in primary rat hippocampus neuron, neuron-like cells derived from hiPSC-CMs and hiPSC-CMs. Representative action potential waveforms for spontaneous action potentials recorded from primary rat hippocampus neurons, week 3 post-transduction neuron-like cells converted from hiPSC-CMs, and nodal-like hiPSC-CMs. Note that action potential waveforms for neuronal cells (primary rat and hiPSC-CM derived neurons) are more similar than nodal hiPSC-CMs.

SUPPLEMENTAL TABLES:

Supplemental Table 1: Qualitative and quantitative criteria for classification of hiPSC-CMs in three major cardiac cell types.

**Nodal-like (N-like):**
▪ Always generated spontaneous APs
▪ Exhibits a more depolarized MDP
▪ A prominent phase 4 depolarization
▪ Slower maximum rate of rise
▪ Shortest APD
▪ APD90/APD50: 1.4-1.7
**Atrial-like (A-like):**
▪ Triangular AP profile
▪ Absence of a prominent plateau phase
▪ A negative MDP (< -50 mV)
▪ More hyperpolarized MDP/RMP
▪ A faster rate of rise
▪ Intermediate APD
▪ APD90/APD50: > 1.7
**Ventricular-like (V-like):**
▪ A negative MDP (< -50 mV),
▪ A rapid AP upstroke,
▪ A long plateau phase,
▪ APA > 90 mV
▪ APD90/APD50: < 1.4

Supplemental Table 2: Summary of action potential parameters of hiPSC-CMs at day 30-32 day of differentiation.

iPSC-CMs	% of cells (n)	Beating rate (bpm)	MDP (mV)	Overshoot (mV)	APA (mV)	APD90 (ms)	APD70 (ms)	APD50 (ms)	V_{max} (V/Sec)
**Day 22-26 (*n* = 24)**									
Nodal-like	13 (3)	76 ± 6	-47.1 ± 1.2	25.7 ± 2.2	72.8 ± 3.5	253.6 ± 27.3	209.1 ± 20.1	175.4 ± 15.0	5.66 ± 1.2
Atrial-like	29 (7)	53 ± 7	-57.7 ± 1.0	39.4 ± 1.1	97.0 ± 1.5	288.0 ± 16.0	212.4 ± 13.0	169.0 ± 14.0	24.0 ± 2.3
Ventricular-like	58 (14)	52 ± 8	-57.1 ± 1.5	44.0 ± 1.0	101.0 ± 2.0	333.1 ± 16.6	293.0 ± 16.0	253.7 ± 17.0	28.0 ± 2.5

BPM: Beat Per Minute
MDP: Maximal Diastolic Potential
APA: Action Potential Amplitude
APD_{50}: Action Potential Duration at 50% repolarization
APD_{70}: Action Potential Duration at 70% repolarization
APD_{90}: Action Potential Duration at 90% repolarization
Vmax: Upstroke Velocity

SUPPLEMENTAL MOVIES:

Supplemental Movie 1: Time-lapse video of transformation of mouse PSC-CMs into neurons

Time-lapse microscopy of BAM lentivirus-transduced eGFP+ CMs for three days following Dox treatment documents the step-wise transition of Nkx2-5-eGFP^{+} mESC-CMs into induced neurons.
